# Host responses to concurrent combined injuries in non-human primates

**DOI:** 10.1186/s12950-017-0170-7

**Published:** 2017-11-02

**Authors:** Matthew J. Bradley, Diego A. Vicente, Benjamin A. Bograd, Erin M. Sanders, Crystal L. Leonhardt, Eric A. Elster, Thomas A. Davis

**Affiliations:** 1 Department of Regenerative Medicine, Naval Medical Research Center, 503 Robert Grant Avenue, Silver Spring, MD 20910 USA; 2 Department of Surgery, Uniformed Services University of the Health Sciences-Walter Reed National Military Medical Center, Bethesda, MD 20184 USA

**Keywords:** Non-human primate, Trauma, Immune response, Military

## Abstract

**Background:**

Multi-organ failure (MOF) following trauma remains a significant cause of morbidity and mortality related to a poorly understood abnormal inflammatory response. We characterized the inflammatory response in a non-human primate soft tissue injury and closed abdomen hemorrhage and sepsis model developed to assess realistic injury patterns and induce MOF.

**Methods:**

Adult male Mauritan Cynomolgus Macaques underwent laparoscopy to create a cecal perforation and non-anatomic liver resection along with a full-thickness flank soft tissue injury. Treatment consisted of a pre-hospital phase followed by a hospital phase after 120 minutes. Blood counts, chemistries, and cytokines/chemokines were measured throughout the study. Lung tissue inflammation/apoptosis was confirmed by mRNA quantitative real-time PCR (qPCR), H&E, myeloperoxidase (MPO) and TUNEL staining was performed comparing age-matched uninjured controls to experimental animals.

**Results:**

Twenty-one animals underwent the protocol. Mean percent hepatectomy was 64.4 ± 5.6; percent blood loss was 69.0 ± 12.1. Clinical evidence of end-organ damage was reflected by a significant elevation in creatinine (1.1 ± 0.03 vs. 1.9 ± 0.4, p=0.026). Significant increases in systemic levels of IL-10, IL-1ra, IL-6, G-CSF, and MCP-1 occurred (11-2986-fold) by 240 minutes. Excessive pulmonary inflammation was evidenced by alveolar edema, congestion, and wall thickening (H&E staining). Concordantly, amplified accumulation of MPO leukocytes and significant pulmonary inflammation and pneumocyte apoptosis (TUNEL) was confirmed using qRT-PCR.

**Conclusion:**

We created a clinically relevant large animal multi-trauma model using laparoscopy that resulted in a significant systemic inflammatory response and MOF. With this model, we anticipate studying systemic inflammation and testing innovative therapeutic options.

## Background

Multi-organ failure (MOF) in patients following complex trauma remains a significant cause of morbidity and mortality [1]. The lungs and the kidneys are two critical organs implicated in MOF and represent a major source of the associated morbidity and mortality after trauma [2–4]. With an incidence of over 10%, acute lung injury leading to acute respiratory distress syndrome (ARDS) has been associated with longer hospital stays, more days of mechanical ventilation, increased complications, and a three-fold increase in mortality compared to trauma patients without ARDS [5, 6]. Likewise, acute kidney injury (AKI) has been found to occur in over 30% of critically ill trauma patients, with 10% of those requiring renal replacement therapy, and has been identified as an independent predictor of mortality [4, 7, 8].

The pathophysiology behind MOF is related to hemorrhagic shock with ischemia reperfusion injury and massive tissue injury. These traumatic events induce abnormal local and systemic inflammatory responses triggered by the innate immune response involving the production and release of excessive pro-inflammatory mediators [9, 10]. Much of what we know in regards to the pathophysiological mechanisms and the maladaptive immune response has come from basic science research using various animal models. Small and large animal models have established the foundation for understanding the physiologic, cellular, immunologic, and molecular responses to hemorrhagic shock and for the development of interventions designed to improve the clinical outcome of trauma patients. However, these animal models vary widely and have translational limitations given that they fail to replicate human physiologic and immunologic responses to trauma and hence promising pre-clinical immunomodulatory agents have not demonstrated significant clinical benefit. These discordant results are thought in part due to variations between small animal species and human response to inflammatory stimuli as demonstrated by Seok et al. and Mestas et al [11, 12]. Larger animals, such as swine, have also been poor models of injury because they do not physiologically or immunologically respond to trauma in the same way as humans [13–17]. Additionally, most models fail to create a realistic injury pattern as seen in a multi-trauma patient. These animal models often involve an open abdominal hemorrhage component via laparotomy, which not only removes the physiologic effect of tamponade but also alters the immune response associated with intraperitoneal blood [18–20].

The ideal MOF model on which to preclinically test immunomodulatory strategies should address these issues by selecting an appropriate large animal model that most closely resembles the human genetic, immunologic, and physiologic responses to polytraumatic injury. The model should also include severe enough injury to simulate a clinically multi-injured, “multi-hit” casualty which predisposes the experimental animal to MOF. In addition, the pre-hospital oxygen debt phase as well as subsequent resuscitation should mirror clinical situations so as to re-create the expected ischemia reperfusion injury and targeted goal directed resuscitation. Finally, measurable targets of distant end organ injury in this model such as inflammatory changes in the lung and alterations in renal function should be identified such that the impact of immunomodulatory intervention could be evaluated throughout the experiment. Our objective was to characterize the physiologic insult and inflammatory response in a non-human primate (NHP) soft tissue injury and closed abdomen hemorrhage and sepsis model. We hypothesized that the NHPs exhibit similar responses following polytraumatic injury when compared to humans. We report that this model reflects the degree of complexity and severity of acute blast-related dismounted combat polytrauma (which can include intra-abdominal injuries, both solid and hollow viscus organs, and soft tissue injuries) without the addition of extremity amputations [21, 22]. Our results provide information relating to key genes associated with acute lung injury and pulmonary-induced inflammation in response to polytraumatic injury, which could serve as new diagnostic and prognostic indicators and biomarkers for clinical assessment as well as identification of new molecular targets for drug development. Moreover, we strongly believe that this NHP model should be used judiciously for studying critically complex mechanistic and translational data (risk to benefit results) prior to evaluating candidate therapeutic approaches applied to patients in clinical trials.

## Methods

### Animals

**Fig. 1:**
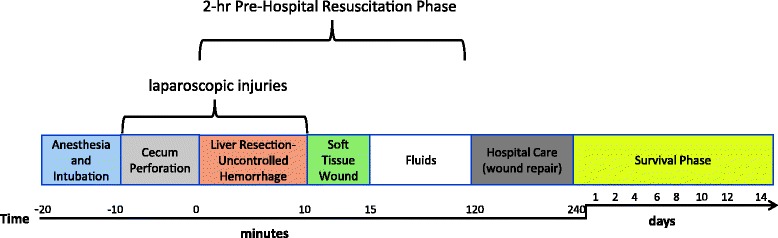
Schematic showing the time frame of traumatic injury with the various post-injury phases

The study protocol was reviewed and approved by the Walter Reed Army Institute of Research/Naval Medical Research Center (NMRC) Institutional Animal Care and Use Committee (#13-OUMD-32) in compliance with all applicable Federal regulations governing the protection of animals in research. Adult male (7.3kg ± 0.15) Mauritan Cynomolgus Macaques (*Macaca fascicularis;* Worldwide Primates, Miami, Florida) were used. NHPs were quarantined for approximately 45 days to acclimate to the animal facility. During that time, they were allowed free access to feed and water. The animals had free access to food and water prior to the start of the experiment; however, oral nutrition was withheld the night prior to surgery (12 hours) to prevent aspiration during anesthesia. Tissues from age-matched uninjured control NHPs (*n* =5) were obtained from archived biobank repositories collected under previous studies. A schematic of the protocol schedule is depicted in Fig. 1.

### Polytrauma model

#### a. Pre-procedure care

On the morning of surgery, an intramuscular injection of Telazol (2-8mg/kg) was given for sedation. This was followed by cannulation of the saphenous vein for initial intravenous access. Mask ventilation with isoflurane was used to facilitate endotracheal intubation with a cuffed endotracheal tube under laryngoscopic guidance.

Anesthesia in the surgery suite was maintained with isoflurane on 21-25% medical air via Datex Ohmeda S/5 ADU Carestation. After intubation, animals were monitored using ECG, pulse oximeter, end tidal carbon dioxide (ETCO_2_), and a rectal temperature probe. Foley bladder catheterization was performed to monitor and quantify urine output. Adjustments in ventilation were made to maintain pCO2 between 35-42 mmHg to avoid hyper- or hypoventilation, 12-15 breaths/minute; tidal volume 5-10 ml/kg; and fraction of inspired oxygen (FiO_2_) 0.21(Apollo /Drǟger Medical, Telford, PA).

#### b. Injury phase

All animals were shaved, prepped with a chlorhexidine/alcohol-based solution, and draped in the standard fashion. A right central venous line (CVL) and arterial (A-line) were placed into the femoral vein and artery, respectively, via a femoral cut-down method. A tunneled 6F vascular access port (PORT-A-CATH, Smiths-Medical, Dublin, OH) was used for long-term venous access. The common femoral artery was cannulated utilizing a 22-gauge angio-catheter (Cordis, Johnson & Johnson) and connected to a hemodynamic monitoring system (Philips IntelliVue MP70, Philips Electronics North America Corporation, Andover MA) for continuous monitoring of arterial pressures and lab draws. Both catheters were secured in place with sutures to prevent dislodgement during the remainder of the procedure.

After placement of the vascular catheters, the intra-abdominal injuries were created using laparoscopic instruments. Initial entrance into the peritoneal cavity was obtained via the Hasson technique. An infra-umbilical incision was made to accept the size of a 12mm trocar (ENDOPATH XCEL, Ethicon, Somerville, NJ), which was inserted into the peritoneal cavity under direct vision. The abdominal cavity was insufflated with CO_2_ to achieve an intra-abdominal pressure of 10 – 12 mmHg allowing for adequate visualization of the intra-abdominal organs. This pressure was constantly monitored and adjusted as needed throughout the procedure. Two additional 5mm trocars were placed under direct visualization, one in the left hypogastric region and one in the right hypogastric region.

The cecum was identified and an approximate four-centimeter perforation was made on the anti-mesenteric side, at least one centimeter away from the terminal ileum, via a laparoscopic shear device. Fecal material was allowed to flow out from the perforated cecum to induce intra-abdominal sepsis. Attention was then directed to the liver. The left lobe of the liver was identified and measured with a sterile plastic ruler in the cranio-caudal aspect using atraumatic graspers to retract the lobe laterally. Approximately 60% of the length of this lobe was then rapidly resected with laparoscopic shears to create a non-anatomic resection with uncontrolled hemorrhage. Once the liver injury was completed the CO_2_ was immediately evacuated from the peritoneal cavity, all laparoscopic ports were removed, and the port sites were temporarily closed with 2-0 nylon sutures to achieve abdominal wall continuity. The initiation of the liver injury was considered the beginning of the pre-hospital phase and referred to as time zero minutes (T=0).

After the laparoscopic injuries were created, the animal’s right flank was sterilely prepared and a circular standardized (18.85cm^2^) full-thickness soft tissue injury was created with electrocautery. The area was excised down to the level of the muscle fascia removing the skin and subcutaneous tissue. Injury severity score as a result of these injuries was calculated as 21 meeting criteria for severe trauma.

#### c. Hemorrhage phase/Pre-hospital care

Fifteen minutes after initiation of the liver injury, pre-hospital treatment commenced with normal saline resuscitation (up to a total pre-hospital fluid volume of 20cc/kg), according to Advanced Trauma Life Support (ATLS) guidelines [23]. Hemodynamic parameters were monitored during this time. To simulate a prolonged transport time, the pre-hospital phase lasted 120 minutes (T=120). Anesthetic gas was adjusted during the injury phase to minimize the anesthetic effects on blood pressure while ensuring adequate sedation.

#### d. Resuscitation phase/Hospital care

At T = 120 minutes the hospital phase was initiated and a midline laparotomy incision from the xiphoid process to just below the umbilicus. The incision was carried through the peritoneum to gain access into the peritoneal cavity. The liver was exposed and liver packing was performed. Once hemostasis was achieved the liver was repaired by over-sewing the cut edge with chromic sutures and cauterizing the raw, exposed parenchyma. Intra-peritoneal blood was evacuated via pre-weighed laparotomy pads for measurement and subsequently weighed in order to formally quantify blood loss. After control and repair of the liver injury, attention was then directed at repairing the cecal perforation. The inflamed edges of the cecal injury were resected with metzenbaum scissors, and a two-layered intestinal repair was performed. Full thickness apposition with 3-0 vicryl sutures was performed followed by imbricated seromuscular sutures of 3-0 silk. All intra-abdominal fecal contamination was removed and the peritoneum irrigated with warm normal saline until the effluent was clear. The abdomen was then closed in two layers with running sutures and the previously placed laparoscopic trocar sites were re-approximated with absorbable suture.

To mimic clinical practice during the hospital phase, the intra-operative resuscitation was targeted to physiologic parameters during the hospital phase. Parameters including mean arterial pressure (MAP) < 40mmHg, base excess > -4, and/or lactate < 4, decreased urine output, and hemoglobin < 7 gm/dL. Crystalloid fluid resuscitation was limited to 40cc/kg during the operative phase with primary hemodynamic support and correction of perfusion deficit was provided by whole blood. This blood was type-specific and was administered through a leuko-reducing filter. A single dose of broad-spectrum antibiotics was given at the initiation of the laparotomy.

After completion of the abdominal portion of the protocol the soft tissue wound was covered with a sterile wound dressing (Hydrofera BLUE®, Hollister Wound Care, Libertyville, IL). The arterial catheter was removed at conclusion of the procedure and the arteriotomy repaired with an interrupted 6-0 prolene suture. The venous port was tunneled in a subcutaneous fashion onto the lateral thigh. Then the wound was closed in layers with absorbable sutures, and dressed with Dermabond (Ethicon, Cincinnati, OH). Following this, the animal was placed in a protective jacket (Lomir Biomedical inc, Quebec, Canada), emerged from anesthesia, and extubated.

#### d. Survival phase

After extubation, the animals were taken to an “intensive care unit” setting where they were recovered under direct observation by research and veterinary medical staff. Animal vital signs and urine output were monitored every 4 hours for the first 72 hours. Evidence of hypotension or low urine output prompted laboratory evaluations and goal directed resuscitation as described above. Additionally, on post-operative day (POD) 1, 2, and 3 animals received an infusion of normal saline (20mL/kg) via the femoral vein mediport. If the NHP was found to be anemic (Hgb <7gm/dL), a blood transfusion (10mL/kg) was administered. Animals were allowed fluids and returned to a regular diet as deemed appropriate by the research team and veterinary medical staff. Pain medication in the form of a sustained release transdermal fentanyl patch [25mcg/hour (hr)] and buprenorphine (0.01-0.03 mg/kg intramuscular (IM) every 6 hours as needed) was administered in the post-operative period. The soft tissue wound dressing was changed every five days. Once recovered and tolerating an oral diet, the animals were returned to their single-caged group housing rooms. Euthanasia occurred at the veterinarian’s discretion or if the animals demonstrated signs of severe disability, severe infection, uncontrolled pain, or showed signs of laboratory or clinical signs of deterioration and failed to respond to resuscitation. At the end of the survival period (post-operative day 14) euthanasia and necropsy were performed.

### Laboratory analysis

Blood samples were collected at 0, 15, 30, 60, and 120 minutes during the hemorrhage period, at 240 minutes during the resuscitation period, as needed in the post-operative setting and daily thereafter. Blood was analyzed for complete blood cell count, arterial blood gas, and basic metabolic profile. In addition, the serum profiles for a panel of 14 cytokines and chemokines (IL-6, IL-1ra, IL-10, G-CSF, MCP-1, IL-1β, IFN-γ, TNFα, GM-CSF, IL-15, MIP-1α, IL-8, IL-2, TGFα) was determined using multianalyte bead based profiling (Luminex; NHP 14-plex; Millipore, Billerica, MA).

### Lung mRNA gene transcript analysis

Total RNA was extracted from freshly isolated lung tissue using standard trizol-chloroform-ethanol extraction procedures. RNA’s were resuspended in 15 μl of 10 mMTris buffer, pH 7.5. Sample purity, quantity, and quality was assessed by determining the A_260/280,_ A_260/230_ ratio on an Nanodrop Spectrophotometer (NanoDrop Technologies Inc. Wilmington, DE) and by measuring 28S/18S ribosomal RNA ratio and RNA Integrity Number (RIN) using an Agilent 2100 BioAnalyzer (Agilent Technologies Inc. Santa Clara, CA). All Agilent RNA integrity values were > 8.5. Reverse transcription was performed with Roche 1^st^ Strand Synthesis kit (Roche Diagnostics Corporation, Indianapolis, IN). Briefly, 2.5 μg of RNA sample was added to a master mix containing 1x reaction buffer, 5 mM MgCl_2_, 1 mM deoxynucleotide mix, 6.4 μg random primers, 100 units RNase inhibitor, and 40 units AMV reverse transcriptase. 10 mMTris buffer, pH 7.5 was used to reach 40 μl final reaction volume. Then, final reaction mixture was subjected to a single reverse-transcription cycle of 25°C for 10 minutes (min), 42°C for 60 min, 99°C for 5 min, and 4°C for at least 10 min. Real-Time quantitative PCR (RT-PCR) was performed for mRNA gene expression profiling. Quantitative real-time polymerase chain reaction (qRT-PCR) was performed using the ABI Prism 7900HT Sequence Detection System (Applied Biosystems, Foster City, CA). Custom designed TaqMan® Low Density Array (TLDA) cards (Applied Biosystems) were used to assess gene expression of 488 key transcripts for inflammation, apoptotic and lung injury mediators. Gene targets were selected based on an extensive review of the literature for well validated gene expression markers and the availability of “Assay of Demand” commercial primers (Applied Biosystems). The set of TLDA cards were comprised of 491 individual target assays (including 3 housekeeping genes 18s, beta actin, and GAPDH); respective forward and reverse primers and a dual labeled probe (5’-6-FAM; 3’-MGB). Amplification parameters were as follows: one cycle of 50^o^C for 2 min and 95^o^C for 10 min followed by 40 cycles of 95^o^C for 30 seconds and 60^o^C for 1 min. RT-PCR data was analyzed using the Sequence Detection System version 2.1 included with the ABI Prism 7900HT SDS and Microsoft Excel. The threshold was manually set and the baseline was set automatically to get the threshold cycle (C_t_) value for each target. Beta actin ribosomal RNA was used as an endogenous housekeeping control gene for normalization. Relative gene expression between normal lung tissue (*n* = 5) and that collected from injured nonsurvivors (range 5.5hr-POD3; *n* =5) was determined using the comparative C_t_ method (2^-ΔΔCt^). Results are expressed as the mean difference in relative fold gene expression. Transcription of a particular gene transcript was considered to be differentially up- or down regulated if it was differentially expressed by at least 2-fold when compared with the expression level in normal lung tissue Assays with C_t_ values greater than 35 cycles were excluded from analysis.

### Histological analysis and myeloperoxidase (MPO) staining

Samples of lung tissue from three non-injured controls (obtained from archived historical samples) and six experimental animals that expired between 5.5 and 30-hours post-injury were fixed in 10% formalin and then embedded in paraffin. Tissue blocks were cut into 5μm step sections, placed onto glass slides and stained with hematoxylin and eosin (H&E), dehydrated, and mounted. Lung sections were evaluated using light microscopy by a pathologist blinded to the mode of injury and clinical outcome. Evidence of lung injury changes consistent with ARDS were assessed using the following criteria: (1) cellular infiltration, (2) alveolar congestion/edema, (3) alveolar hemorrhage, (4) alveolar collapse and (5) septal membrane thickening. The severity of each of these pathological features was evaluated by a score indicating 0 as absent/none, 1 as mild, 2 as moderate, and finally 3 for severe injury. Compilations of these values obtained from individual pathological features represent the lung injury score.

To measure neutrophil infiltration in lungs we performed immunohistochemistry to detect myeloperoxidase (MPO) positive leukocytes. Formalin-fixed lung tissue embedded in paraffin was serially sectioned (5μm) onto glass slides. Tissue sections were then deparaffinized in xylene, and rehydrated in a graded series of ethanol. Antigen retrieval was achieved by heated by heating slides in citric acid buffer (pH 6.0) for 30 min. After antigen-retrieval, slides were blocked with 3% bovine serum albumin (BSA) then incubated with 1.5% hydrogen peroxide for 10 min, to block endogenous peroxidase activity. Sections were next incubated overnight at 4^o^C with a 1:40 dilution of a rabbit polyclonal antibody to MPO (SC 33596, Santa Cruz Biotechnology, Inc., Dallas, TX). Vectastain ABC HRP reagent and DAB kit (Vector Laboratories, Burlingame, CA) were used to detect the immunohistochemical reaction. Slides were counterstained with 4′, 6-diamidino-2-phenylindole. Microscopic examination, image acquisition and representative photomicrographs were taken using a brightfield /fluorescence microscope (Olympus IX73 Imaging System with Olympus DP73 Universal Camera, Olympus, Pittsburgh, PA). MPO-positive staining cells were counted in 10 random visual fields/section at × 200 magnification and averaged.

### Fluorescent TUNEL assay for detecting apoptotic cells

Terminal deoxy-nucleotidyl transferase (TdT)-mediated dUTP nick end-labeling (TUNEL) was conducted on collected lung tissue from three non-injured controls and four experimental NHPs by using the in situ Cell Death Detection kit from Roche Applied Science (Indianapolis, IN) as previously described [24]. Briefly, paraffin-embedded lung sections were deparaffinized and permeabilized with 0.1 M sodium citrate buffer, pH 6.0 at 65 °C for 1 hr. Next, sections were incubated with the TUNEL reaction buffer for 1 hr at 37 °C in a humidified chamber. Sections were incubated with 4’,6-diamidino-2-phenylindole (DAPI) to allow visualization of cell nuclei (Molecular Probes, Eugene, OR). Immunofluorescence was assessed using a Zeiss LSM 710 confocal microscope under oil-immersion objectives. The number of TUNEL-positive cells was identified by a pathologist blinded to the mode of injury and clinical outcome using Metamorph software (Molecular Devices Corp.) and expressed as the number of TUNEL-positive cells per high-power field, with 10 random fields per slides counted.

### Statistical analysis

Statistics were performed in STATA Version 13 (StataCorp, College Station, TX). Percent blood loss was calculated using the formula: [blood loss volume (ml)/total estimated blood volume (TEBV)] x 100. TEBV was calculated based on 5.4% of the animal’s weight according to previously published data on blood volume in cynomolgus macaques [25]. Percent hepatectomy was calculated using the formula: [liver cut length (cm)/full liver length (cm)] x 100.

## Results

**Fig. 2:**
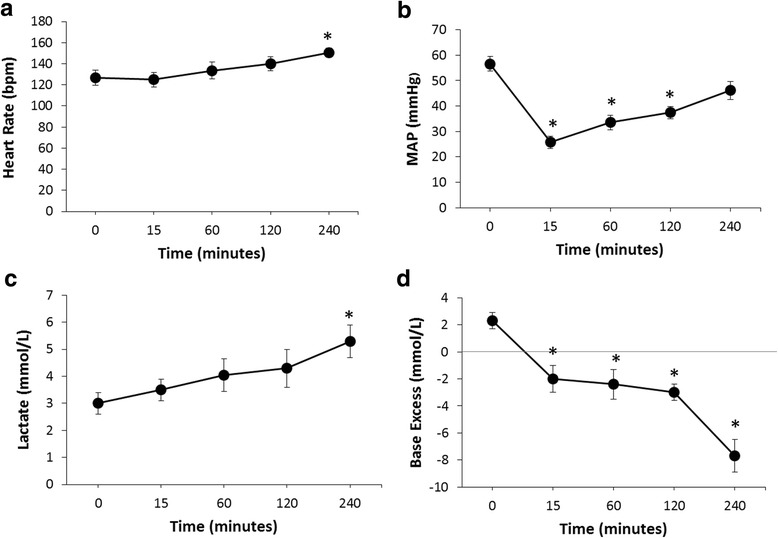
(**a**) Time course of heart rate (HR), (**b**)  mean arterial pressure (MAP), (**c**)  blood lactate level and (**d**) base excess level measurements at different time points for the first 4 hr following polytraumatic injury and during the early reperfusion/ resuscitation and recovery phase. Each point represents the mean ± SEM of surviving NHPs. * *P*<0.05 indicates a significant difference from T0 (pre-hemorrhage)

Twenty-one animals with a mean weight of 7.3kg ± 0.15 underwent the experimental protocol. Mean percent hepatectomy for the group was 64.4 ± 5.6, while percent blood loss was 69.0 ± 12.1. Significant clinical and laboratory parameters are listed in Table 1. Evidence of end-organ damage as a result of the induced injury was reflected by a statistically significant elevation in creatinine from baseline (1.1 ± 0.03 vs. 1.9 ± 0.4, p = 0.026). Likewise, significant changes in lactate (3.0 ± 0.3 vs. 6.3 ± 0.7, *p* < 0.001) and base excess (2.2 ± 0.6 vs. -8.6 ± 1.2, *p* < 0.001) occurred during the study indicating shock and anaerobic metabolism. Trends of heart rate and MAP as well as lactate and base excess over the experimental period for the animals are displayed in Fig. 2. Heart rate and lactate gradually increased over the study time period while MAP decreased rapidly hitting a nadir at the 15-minute post-injury time period. Base excess diminished over time resulting in a significant deficit at the end of the pre-hospital time period.

**Table 1:** Clinical and Laboratory Parameters at Baseline and During the Hours Following Injury

Weight (Kg)	7.3 ± 0.15	Initial Heart Rate	128.2 ± 6.8
Blood Loss Amount (mL)	284.0 ± 49.0	Maximum Heart Rate	161.3± 5.0
Percent Blood Loss	69.0 ± 12.1	Starting MAP	56.0 ± 2.5
Percent Hepatectomy	64.4 ± 5.6	Lowest MAP	24.4 ± 1.8
Blood Transfusion Volume (mL)	100.2 ± 20.4	Initial pH	7.40 ± 0.01
Initial Hgb (g/dL)	12.3 ± 0.2	Final pH	7.35 ± 0.03
Final Hgb (g/dL)	10.9 ± 0.4	Initial Lactate (mmol/L)	3.0 ± 0.3
Initial WBC	9.7 ± 1.0	Maximum Lactate (mmol/L)	6.3 ± 0.7**
Maximum WBC	22.8 ± 1.4	Initial Base Excess (mmol/L)	2.2 ± 0.6
Initial Creatinine	1.1 ± 0.03	Maximum Base Excess (mmol/L)	-8.6 ± 1.2**
Maximum Creatinine	1.9 ± 0.4*		

Values listed as mean ± Standard Deviation. Hgb = Hemoglobin; WBC = white blood cell count; MAP = mean arterial pressure. *p <0.05 **p<0.001

**Fig. 3:**
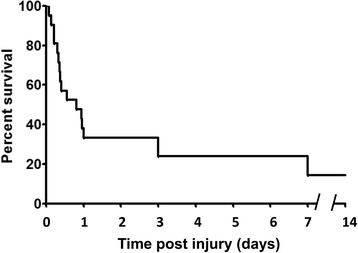
Kaplan-Meier curve for 14-day survival of NHPs after polytraumatic injury (*n* = 21)

Mean survival in days was 2.62 ± 1.1 with the majority of mortalities occurring between 5.5 hr postoperatively and POD-3. A Kaplan-Meier survival curve is depicted in Fig. 3. Overall, early mortality, prior to POD 14, was 86%. Etiology of early mortality included intra-operatively (*n* = 3) due to massive hemorrhage and euthanasia in the immediate post-operative period prior to POD-1 (n=8) associated with hemorrhagic shock, cardiovascular collapse, or failure to wean from ventilator support. Euthanasia on POD-3 (*n* = 5) was performed for respiratory failure as evidenced by tachypnea and oxygen desaturations and renal failure with oliguria despite targeted resuscitation. Finally, euthanasia performed on POD-7 (*n* = 3) was performed for progressive signs of distress associated intra-abdominal sepsis, which was confirmed on necropsy.

**Fig. 4:**
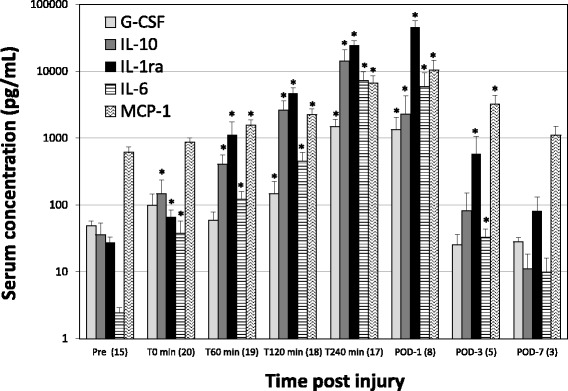
Comparison of serum G-CSF, IL-10, IL-1ra, IL-6 and MCP-1 (CCL2) levels at various time points following polytraumatic injury. Results are expressed as the means ± SE of the number of survived NHPs noted in parentheses each below time point. * *P*<0.05 indicates a significant difference from T0 (pre-hemorrhage)

The acute systemic inflammatory response measured by key cytokines and chemokines is displayed in Fig. 4. Significant increases from baseline (measured two-weeks prior to experimental protocol) occurred with IL-10, IL-1ra, IL-6, G-CSF, and MCP-1. IL-6 and IL-10 reached a maximum by 240 minutes after initiation of the liver injury while IL-1ra and MCP-1 levels continued to increase reaching an apex by POD-1. Of the cytokines measured IL-6 and IL-1ra had the highest fold increase from pre-injury baseline values with IL-1ra reaching > 1600-fold by POD-1 and IL-6 surpassing a 2900-fold increase by T-240 (Table 2).

**Table 2:** Fold increase compared to pre-injured values

	T240	POD-1
G-CSF	30.3	27.4
IL-10	39.7	63.8
IL-1ra	886	1658.1
IL-6	2985.6	2431.1
MCP-1	10.8	16.8

**Fig. 5:**
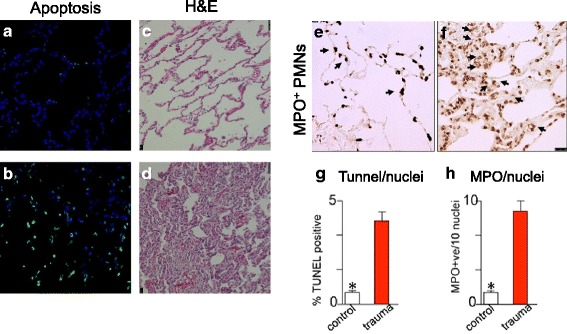
Comparison of TUNEL, hematoxylin and eosin (H&E), and myeloperoxidase (MPO) staining in control and experimental NHPs. **a**-**b**: TUNEL staining in control (**a**) and experimental NHPs (**b**) demonstrated a significant difference in pneumocyte apoptosis in the experimental, polytrauma group (**g**). **c**-**d**: H&E staining of control (**c**) and experimental NHPs (**d**) showing increased airspace edema and alveolar wall thickening in the experimental group. **e**-**f**: Comparison of MPO activity in control (**e**) versus experimental NHPs (**f**) revealed a significant difference accumulation of MPO^+^ activated neutrophils in the polytrauma arm (**h**)

We next evaluated the degree of local lung tissue injury. Excessive pulmonary inflammation was present as evidenced by alveolar edema, alveolar congestion, and alveolar wall thickening on H&E staining (Fig. 5c & d). In addition, compared to control NHPs the early euthanasia NHPs demonstrated amplified accumulation of MPO^+^ leukocytes (Fig. 5e, f, & h) into the lungs as well as significant pneumocyte apoptosis based on TUNEL staining (Fig. 5a, b, & g).

Based on the preceding observations, we confirmed that nonsurvivors mortalities were associated with the activation of a large number of pulmonary proinflammatory triggers involved in leukocyte mediated inflammation responses and acute inflammatory lung injury/damage. In a small cohort of nonsurvivors (5.5 hr- POD-3, *n* =5), we analyzed the differential expression of 378 gene transcripts using PCR (qRT-PCR) using a custom-made TaqMan gene expression low density microarray consisting of transcriptomic targets described to be associated with acute lung injury. Gene expression profiling of 488 transcriptomic lung mRNA gene targets associated with acute lung injury (Table 3) found that a total of 81 differentially expressed genes (DEGs) were identified in lung tissue from nonsurvivors, including 71 upregulated and 10 downregulated genes, when compared to expression levels from lung tissue collected from normal non-injured controls (n=5). The up-regulated DEGs (2.1-7798-fold increase) consisted mainly of proinflammatory signaling/neutrophil activation molecules (cytokine and chemokines), and markers of oxidative stress, extracellular matrix deposition, and epithelial cell injury. The down-regulated DEGs (4-30-fold decrease) were associated with apoptotic cell death (c-FOS), Jak-Stat (IL23r) signaling cascades, smooth muscle contraction (HRH1), angiogenesis (VEGF-D/ FIGF), recruitment chemokines for activated T-cells (CXCL11), and leukocyte adhesion receptor expression (CX3CR, CCR8).

**Table 3:** Differential expression of genes from injured lung tissue in comparison control non-injured lung tissue

Accession No.	Gene Symbol	Gene Name	Molecular Function	Fold Change
NM_001047130	IL2	Interleukin 2	T-cell activation and proliferation, B-cell, NK cell, and monocyte activation	7798.12
XM_001093307	SPP1	Secreted phosphoprotein 1	Upregulation of inflammatory cytokines including IL-12 and Interferon Gamma	1139.76
XM_001110657	CXCL14	Chemokine (C-X-C motif) ligand 14	Chemotactic for monocytes	382.36
	MIP-2γ			
XM_001095645	MAL	Mal, T-cell differentiation protein	Endoplasmic reticulum protein likely associated with T-cell signal transduction	257.02
XM_001103106	PRG3	Proteoglycan 3	Stimulates neutrophil respiratory burst and IL-8 production, as well as basophil histamine and leukotriene C4 production.	202.97
XM_001101685	KNG1	Kininogen 1	Part of kallikrein blood coagulation and can be cleaved to produce bradykinin	86.68
XM_001092700	FGF10	Fibroblast growth factor 10	Involved in wound healing, and secreased by alveolar epithelial cells in response to injury.	81.64
XM_001094451	LBP	Lipopolysaccharide binding protein	Acute phase reactant to gram-negative bacteria and facilitates LPS binding to CD14 on monocytes.	63.00
XM_001108113	IFNW1	Interferon, omega 1	Involved in Toll-Like receptor Signaling Pathways and Type I interferon receptor binding.	47.27
XR_092351	LOC100426120	BCL2/adenovirus E18 19 kDa protein interacting protein 3-like	BCL2 – Suppresses apoptosis by regulating mitochondrial membrane permeability and inhibits caspase activity.	46.51
XM_001112015	LTA	Lymphotoxin alpha (TNF superfamily, member 1)	Member of TNF family and mediates various inflammatory, immunostimulatory, and antiviral responses.	31.78
XM_001100604	BNIP2	BCL2/adenovirus E1B 19kDa interacting protein 2	Inhibits cell death	31.56
XM_001110285	BCL2L1	BCL2-like 1	Regulates mitochondrial release of apoptotic factors	15.07
NM_001032946	CCL23	Chemokine (C-C motif) ligand 23	Potent chemotactic factor for monocytes, resting T-cells, and neutrophils.	23.81
	MIP-3			
XM_001107576	IFNA14	Interferon, alpha 14	Binds type interferon receptor and demonstrates anti-viral activity	19.58
XM_001107510	IL1R1	Interleukin 1 receptor, type I	Mediates IL-1 dependent activation of NF-kappa-B and MAPK pathways.	18.46
XM_001087874	LOC699418	Eosinophil lysophospholipase-like	Protecgt eosinophils from being lysed by large amounts of phospholipids	15.98
XM_001117159	IL22	Interleukin 22	Part of IL-10 family and initiates innate immune responses against bacteria in gut and respiratory epithelial cells.	14.93
XM_001110530	S100A8	S100 calcium binding protein A8	Involved in cell cycle progression and differentiation intracellularly as well as neutrophil chemotaxis, proinflammatory, pro-apoptotic, DAMP, and oxidant-scavening	12.67
XM_001108108	IL18RAP	Interleukin 18 receptor accessory protein	Enhances IL-18R signaling though PEDF induced signaling.	12.31
XM_00108022	PROK2	Prokineticin 2	Involved in circadian rhythm and gastrointestinal smooth muscle contraction.	10.89
XM_001113239	IL22RA1	Interleukin 22 receptor, alpha 1	Part of IL-10 receptor complex and induces JAK/STAT pathways as well as anti-angiogenic activity.	10.09
XM_001096915	TNFRSF11B	Tumor necrosis factor receptor superfamily, member 11b	TNF receptor family inhibits apoptosis as decoy receptor for TRAIL.	9.86
XM_001082859	CASP9	Caspase 9, apoptosis-related cysteine peptidase	Pro-apoptotic by through activation of caspase cascade.	9.61
NM_001042733	IL6	Interleukin 6 (interferon, beta 2)	Pluripotent cytokine which is anti-apoptotic and pro-inflammatory in acute phase response	9.28
XM_001104328	ADORA1	Adenosine A1 receptor	Adenosine receptor inhibits adenylyl cyclase	9.27
XM_001085850	BCL2L10	BCL2-like 10 (apoptosis facilitator)	Inhibits apoptosis by preventing mitochondrial cytochrome C release.	8.33
NM_001032875	CCL20	Chemokine (C-C motif) ligand 20	Chemotaxis of dendritic cells, T-cells, and B-cells.	7.60
	MIP-3α			
XM_001083110	PXMP2	Peroxisomal membrane protein 2, 22kDa	Pore-forming activity in peroxisomal membrane	6.54
XM_001108040	BIK	BCL2-interacting killer (apoptosis-inducing)	Accelerates apoptosis	5.34
XM_001117272	LOC721200	Complement component 4A (Rodgers blood group)	Essential for the propagation of classical complement pathway and plays and major role infection diseases and inflammatory processes	5.09
XM_001118232	TNFRSF1A	Tumor necrosis factor receptor superfamily, member 1A	Primary receptor for TNFa and pro-apoptotic.	5.01
NM_001142873	MYC	V-myc myelocytomatosis viral oncogene homolog (avian)	Cell cycle progression and pro-apoptotic	4.97
NM_001047134	IL1R2	Interleukin 1 receptor, type II	Acts as decoy receptor for IL-1 and inhibits the activity of IL-1R1 ligands.	4.93
XM_001082504	IL17RB	Interleukin 17 receptor B	Induces production of IL-8	4.67
XM_001112911	TYMP	Thymidine phosphorylase	Promotes angiogenesis by proliferation of endothelial cells.	4.60
XM_001086840	APAF1	Apoptotic peptidase activating factor 1	Promotes apoptosis by binding cytochrome C and activates caspase cascade.	4.53
XM_001104160	BAG3	BCL2-associated athanogene 3	Anti-apoptotic and binds HSP70/HSC70.	4.36
XM_001107790	TNFRSF10A	Tumor necrosis factor receptor superfamily, member 10a	Receptor for TRAIL and induces apoptosis.	4.29
XM_001090833	CASP7	Caspase 7, apoptosis-related cysteine peptidase	Induces apoptosis	4.20
XM_001093641	IL-1F5	Interleukin 36 receptor antagonist	Inhibits NK-kB activation and prevents IL-36R signaling, and may activate anti-inflammatory pathway	4.05
XM_001109281	BCL2A1	BCL2-related protein A1	Inhibits apoptosis	3.86
XM_001091119	IL1F8	Interleukin 36, beta	Pro-inflammatory signaling through IL-1RL2 and IL-36R, present in epithelial barrers, and stimulates production of IL-6 and IL-8 and various chemokines.	3.85
XM_001100700	BLNK	B-cell linker	Involved in B-cell receptor signaling and activation of ERK/EPHB2, MAP, p38, AP1, NF-kB, and PLCG1 and 2 activation.	3.78
XM_001084983	DAPK1	Death-associated protein kinase 1	Mediates IFNg signaled cell death	3.59
XM_001099305	MGLL	Monoglyceride lipase	Metabolizes free fatty acids and glycerol, contributes to endocannabinoid signaling.	3.56
XM_001090430	ERBB2	V-erb-b2 erythroblastic leukemia viral oncogene homolog 2, neuro/glioblastoma derived oncogene homolog (avian)	Part of epidermal growth factor receptor family and enhances activation of MAP-K and PI3K signaling pathways.	3.55
NM_001032917	IL2RA	Interleukin 2 receptor, alpha	Part of IL-2 receptor family involved in mediating immune tolerance through regulatory T-cell activity.	3.50
XM_001092296	LEFTY2	Left-right determination factor 2	TGF-beta receptor ligand and activates SMAD signaling pathway.	3.14
XM_001090570	BAK1	BCL2-antagonist/ killer 1	Mediates mitochondrial release of cytochrome C and accelerates apoptosis.	3.30
XM_001089600	MAPK1	Mitogen-activated protein kinase 1	MAP kinase signaling transduction pathway involved in cell growth, adhesion, survival, and differentiation.	3.04
XM_001086237	BCL2L11	BCL2-like 11 (apoptosis facilitator)	Induces apoptosis through caspase mediated pathway	3.01
XM_001095097	CSF3	Colony stimulating factor 3 (granulocyte)	Granulocyte stimulating factor involved in production, differentiation, and function of Granulocytes.	3.00
	G-CSF			
NM_001032846	IL7	Interleukin 7	Pro-inflammatory cytokine involved in both B-cell differentiation and Th1 response.	2.96
XM_001117239	CRP	C-reactive protein, pentraxin-related	Acute phase reactant, part of pentaxin family and involved in agglutination, bacterial capsular swellilng, complement fixation, and phagocytosis.	2.83
XM_001104958	IFNK	Interferon, kappa	Type I interferon family and regulates immune cell function.	2.81
XM_001103515	PTX3	Pentraxin 3, long	Part of pentraxin protein family induced in inflammatory response and promotes fibrocyte differentiation, and involved in regulating inflammation and complement activation.	2.76
XM_001099255	SERPINA1	Serpin peptidase inhibitor, clade A (alpha-1 antiproteniase, antitrypsin), member 1	Proteolytic activity against plasmin, thrombin, trypsin, chymotrypsin, plasminogen activation, and decreases coagulation time.	2.72
XM_001101015	TNFSF18	Tumor necrosis factor receptor superfamily, member 18	Co-stimulator for T-cell activation and proliferation.	2.68
XM_001083745	OSMR	Oncostatin M receptor	Involved in IL-31 signaling and activation of STAT pathway.	2.62
XM_001108593	LIF-L	Leukemia inhibitory factor-like	Involved in cell death signaling pathway	2.58
XM_001100017	CXCR5	Chemokine (C-X-C motif) receptor 5	Chemoattractant for B-cells	2.56
XM_001106071	IL17B	Interleukin 17B	Pro-inflammatory and induces release of TNFa and IL-1b.	2.52
XM_001090729	INHBB	Inhibin, beta B	Part of TGF-beta family and involved in PEDF induced signaling and TGF-beta signaling pathways.	2.46
XM_001083817	PCGF2	Polycomb group ring finger 2	Negative regulatory of transcription for cell proliferation control, cytokines, chemokines, and chemokine receptors.	2.45
XM_001094967	SELE	Selectin E	Expressed by activated endothelial cells and involved in leukocyte trafficking	2.39
XM_001109474	EPHX2	Epoxide hydrolase 2, cytoplasmic	Involved in xenobiotic metabolism	2.30
NM_001032915	CXCL9	Macrophage migration inhibitory factor (glycosylation-inhibiting factor)	Pro-inflammatory cytokine which counteracts effects of glucocorticoids, but also regulates regulate macrophage activity in host defense.	2.29
	MIF			
XM_001088935	CD70	CD70 antigen-like	Induces proliferation in activated T-cells, and further increases T-cell activation.	2.28
XM_001115079	RPL13A	Ribosomal protein L13A	Part of Gamma Interferon Inhibitor of Translocation complex functions to inhibit inflammatory response.	2.17
NM_001032821	CCL2	Chemokine (C-C motif) ligand 2	Chemotactic for monocytes and basophils, and augments monocyte activity.	2.17
	MCP-1			
XM_001094589	TRAP1	TNF receptor-associated protein 1	Encodes for heat shock proteins and interacts with TNF type I to regulate cellular stress response.	2.13
XM_001103121	LOC718480	Peptidoglycan recognition protein 1-like	Involved in TNF signaling pathway, also displays bactericidal activity toward Gram-positive and bacteriostatic activity activity toward Gram-positive.	2.04
	PGLYRP			
XM_001112492	LOC711637	Regenerating islet-derived protein 3-gamma-like	Bactericidal and bacteriostatic activity towards Gram-negative	0.25
	REG3G			
XM_001084526	CX3CR1	Chemokine (C-X3-C motif) receptor 1	Binds CX3CL1 and mediates both adhesive and migratory functions.	0.25
XM_001088286	HRH1	Histamine receptor H1	Mediates contraction of smooth muscle, and increases capillary permeability.	0.25
NM_001032848	GREM1	Gremlin 1	Inhibits monocyte chemotaxis.	0.23
XM_001101077	FIGF	C-fos induced growth factor (vascular endothelial growth factor D)	Promotes angiogenesis, lymphogenesis, and stimulates proliferation and migration of endothelial cells.	0.21
NM_001032950	CXCL11	Chemokine (C-X-C motif) ligand 11	Induced by IFN gamma, and is chemotactic for activated T-cells by binding CXC receptor 3.	0.21
	I-TAC			
XM_001088781	FOS	FBJ murine osteosarcoma viral oncogene homolog	Involved in cell proliferation and differentiation.	0.17
XM_001093896	IL23R	Interleukin 23 receptor	Mediates T-cell, NK cell, and myeloid cell stimulation.	0.14
XM_001104979	LOC708837	Similar to growth differentiation factor 9 precursor	A member of the transforming growth factor-β (TGF-β) superfamily,involved in ovarian folliculogenesis	0.10
XM_001084047	CCR8	Chemokine (C-C motif) receptor 8	Involved in monocyte chemotaxis	0.03

## Discussion

In this study, we demonstrate the physiologic insult and inflammatory response in a NHP polytraumatic model (liver hemorrhage, cecal and soft tissue injury) using a laparoscopic technique. The combination of hemorrhage, septic insult, and soft tissue injury resulted in a systemic inflammatory response (SIRS) and multiple, end-organ damage manifested by the development of pulmonary compromise, and acute kidney injury (AKI) [26]. These findings are consistent with the clinical picture of severe polytrauma patients, and identified several markers of injury and inflammation associated with multiple organ failure [27]. In addition to the hemodynamic changes and markers of poor tissue perfusion, the inflammatory response, specifically the IL-6 levels seen at 4 hours and on POD-1, is similar to values predictive of MOF in severe trauma patients at similar time points [28]. Therefore, we conclude that this model reflects the degree of complexity and severity of acute polytraumtic injuries observed in both the military and civilian trauma settings [22, 29].

Evidence of trauma induced MOF was seen in the early euthanasia NHPs which manifested signs consistent with pulmonary injury. While there was no post-operative respiratory support or indwelling central venous pressures included to characterize the severity of the lung injury (i.e. PaO2/FiO2 ratio and Pulmonary Capillary Wedge Pressure), there was evidence of significant pulmonary injury by H&E staining, immunohistochemistry, and gene expression data in the early euthanasia NHPs. The stark contrast of NHP lung tissue in early euthanasia NHPs vs. non-injured control NHPs demonstrates increased leukocyte infiltration and activation with increased MPO staining as well as increased apoptosis signaling per the Tunnel staining [30]. While the initial saline resuscitation could have contributed to the pulmonary edema identified, the degree of inflammation and apoptosis is more consistent with injury model as the cause of these findings.

Accumulating evidence suggests that the upregulation of adhesion molecules, the infiltration of neutrophils and the production of mediators they release including cytokines, chemokines, proteolytic enzymes, reactive oxygen species (ROS) and other inflammatory /cytotoxic mediators within the pulmonary microvasculature, are key to the initiation and perpetuation of lung injury following trauma, shock and sepsis [31, 32]. Similarly, we found in our model of polytrauma-induced lung injury/inflammation increased transcriptional activation of NF-κB signaling leading to increased transcription of specific proinflammatory cytokine and chemokine genes and receptors such as IL-2, SPP1, CXCL14 (MIP-2γ), FGF10, LBP, LTA, IFNW1, IFNA14, IL-1r1, IL-18rap, IL-22ra1, CCL23 (MIP-3), IL-22, S100A8 (calgranulin A), IL-6, CCL20 (MIP-3α), IL-1r2, IL-17rb, IL1F8, IL-2ra, IL-2ra, CSF3 (G-GSF), IL-7, TNFSF18, OSMR, IL-17β, INHBB, SELE, CXCL9 (MIF), CD70, CCL2 (MCP-1), and TRAP1. Increased expression of these mediators leads to recruitment and leukocyte-transendothelial migration of neutrohils, monocytes/macrophages and lymphocytes to the inflammatory site and stimulates proinflammatory activation and cellular apoptosis [31, 32]. Consistent with acute alveolar injury and alveolar apopotosis, we found that expression levels of both pro-apoptotic (BLL2L1, CASP9, BIK, TNFRSF1A, MYC, APAF1, TNFRSF10A, DAPK1, BAK1, and LIFL) and anti-apoptotic (BCL2/BNIP2, BNIP2, TNFRSF11B, BCL2L10, and BAG3, BCL2A1) genes were elevated compared with corresponding levels in lung tissue from uninjured control NHPs [33]. The DEGs from the early euthanasia NHPs further support pulmonary inflammatory response with upregulation of ARDS associated genes including CCL2 (MCP-1), CXCL9 (MIF), APAF1, IL1R2, IL6, CASP9, S100A8, LBP, FGF10, Kininogen, and IL2 [34–46]. Animals also developed stage I kidney injury according to the Acute Kidney Injury Network, a collaborate network of intensivists and nephrologists focused on AKI [26].

The use of minimally invasive techniques is critical in development of animal trauma models, given the significantly increased inflammatory response demonstrated from clinical data comparing laparotomy vs. laparoscopic techniques for elective surgery [47, 48]. We further support these findings with the minimal elevation in cytokine markers with anesthesia and laparoscopic instrumentation seen at T=0 compared to baseline. Recently, a swine laparoscopic liver hemorrhage model was published. In this article the authors describe the creation of an 80% hepatectomy to induce hemorrhage [49]. While laparoscopy was used to create a blunt injury with a larger percent hepatectomy, this protocol only involved hemorrhage to a solitary organ system. This hemorrhage alone model did not generate a severe enough inflammatory response, despite an 80% hepatectomy, and survival out to the 14-day period was relatively high at 75%. Likewise, we have shown the feasibility of a laparoscopic method for creating non-survivable, uncontrolled liver hemorrhage NHP model using a 60% left lobe hepatectomy [50]. However, similar to our findings this injury resulted in a less intense inflammatory response compared with our polytraumatic (liver hemorrhage, cecal and soft tissue injury) model. Recently, Sheppard and colleagues evaluated the physiologic, metabolic, coagulopathic and immunologic responses to controlled pressure-targeted hemorrhagic shock alone (non-trauma-induced; 30-60 min with MAPs within 20-24mmHg) in combination with a 15 cm laparotomy incision and femur fracture [51]. Survival within the resuscitation period required inclusion of 5 minutes of closed chest compressions and two intravenous doses of 2 mg epinephrine. Seven of eight animals expired within 24 hours (54% mean total blood loss) and 6 out of 8 animals failed to meet the recovery criteria of consciousness, alertness and mobility. As expected and consistent with our findings, albeit to a lesser extent, the authors demonstrate that polytraumatic injury induced a more robust systemic inflammatory response with greater circulating levels of IL-6, IL-8, IL-10 and MCP-1 in comparison to other injury patterns assessed. Thus, the additions of second and third “hits” are not only clinically relevant but also important in the development of a maladaptive inflammatory response.

In our NHP polytraumatic (liver hemorrhage, cecal and soft tissue injury) model, we selected a pre-hospital time of 120 minutes to mimic a prolonged first-responder treatment and transport stage. We employed saline as the resuscitation fluid given the fact that blood products in austere combat field environments are not routinely available to the medic, or are rarely used, due to supply and logistics [52, 53]. While saline can lead to a non-gapped acidosis, and may have contributed to the acidosis, the animals in this study experienced a gapped acidosis likely from the elevated lactate and correlating with the severity of injury. This situation can occur in both civilian and military settings particularly in the early course of combat when medical assets have not been fully established in theater, and tissue ischemia time, septic insult exposure, hypothermia, and overall oxygen debt can be incurred. In addition, Army Medics, Navy Corpsmen, and Air Force Pararescuemen are frequently working in austere environments with delays in evacuation due to the operational situation. Prior to then Secretary of Defense Gates’ mandate to reduce aeromedical evacuation time to meet the “golden hour” (< 60 minutes to surgical care) rule, transport times in Afghanistan were on average 100 minutes. A study by Morrison and colleagues found prolonged evacuation times (75- 78 minutes) from point of injury to initial surgical treatment even in the later years of the war in Afghanistan with advanced medical transport assets [54].

A reliable and reproducible preclinical non-human primate model that reproduces developmental and gender-dependent responses to acute polytramatic injury is vitally important. Previous authors have demonstrated that, in addition to genetic similarities, NHPs physiologic and immunologic response to trauma is comparable to humans [55–59]. However, these studies have focused on hemorrhage alone without additional intra-abdominal trauma (liver and intestinal) involving sequelae of fecal spillage making these models clinically less applicable. Using our model, we now have a platform to gain a better understanding of the abnormal inflammatory response following polytrauma trauma and hemorrhage. Ultimately, this will allow us to identify modifiable targets of the dysregulated immune response in anticipation of providing therapeutic agents to mitigate the immune system’s effects and improve outcomes.

The model in its current form has several limitations for studying post-traumatic immune dysregulation and organ injury. The authors recognize that analgesia and anesthesia given before and during the injury phase can affect physiologic and potentially immunologic responses to trauma. While we are attempting to recreate a truly clinically relevant model it is unrealistic, unsafe, and unethical to remove all analgesia and anesthesia support from the experimental design. Furthermore, all animals received equivocal doses of medications thus controlling for this confounding factor. In addition, while the early euthanasia NHPs in this model provided vital tissue data demonstrating the pulmonary injury and possible signs of early ARDS, the lack of post-operative ventilator support likely resulted in the early decompensation of these NHPs which may have otherwise gone on to develop a sustained inflammatory response and MOF. The addition of post-operative ventilator support would allow for management of the NHPs more consistent with clinical practice following severe trauma. To accomplish this goal requires resources for training, purchasing, and maintenance of a ventilator(s), as well as prolonged goal directed post-injury critical care management with continuous vital signs, serial blood counts and metabolic panels, and input/output monitoring.

## Conclusion

We demonstrate the physiologic insult and inflammatory response in a NHP polytraumatic model (liver hemorrhage, cecal and soft tissue injury) using a laparoscopic technique. The injury patterns simulate a clinically relevant blunt torso trauma scenario and removed the inflammatory effects induced via a laparotomy. With further development of this model to include post-operative ventilator support, this model would be useful in testing therapies that modulate/prevent various factors of the secondary injury cascade and would improve the translation of preclinical findings into successful clinical treatments directed at mitigating the morbidity and mortality of polytrauma.

## Abbreviations

2^-ΔΔCt^: Comparative C_t_ method
AKI: Acute kidney injury
A-line: Arterial line
ARDS: Adult respiratory distress syndrome
ATLS: Advanced Trauma Life Support
BSA: Bovine serum albumin
C_t_: Threshold cycle
CVL: Central venous line
DAPI: 4’,6-diamidino-2-phenylindole
DEGs: Differentially expressed genes
ET_C_O_2_: End-tidal carbon dioxide
H&E: Hematoxylin and eosin
Hr: Hour
IM: Intramuscular
Min: Minute
MOF: Multi-organ failure
MPO: Myeloperoxidase
NMRC: Naval Medical Research Center
POD: Post-operative day
qRT-PCR: Quantitative real-time polymerase chain reaction
RIN: RNA Integrity Number
ROS: Reactive oxygen species
SIRS: Systemic inflammatory response syndrome
T=0: Time zero minutes
T=120: 120 minutes
TdT: Terminal deoxy-nucleotidyl transferase
TEBV: Total estimated blood volume
TLDA: TaqMan® Low Density Array
TUNEL: Terminal deoxy-nucleotidyl transferase (TdT)-mediated dUTP nick end-labeling
